# Expression of matrix metalloproteinase-1, -2 and -3 in squamous cell carcinoma and actinic keratosis

**DOI:** 10.1038/sj.bjc.6690468

**Published:** 1999-06

**Authors:** R Tsukifuji, K Tagawa, A Hatamochi, H Shinkai

**Affiliations:** Division of Dermatology, National Chiba Hospital, 4-1-2, Tsubakimori Chiba 260-0042, Japan; Department of Dermatology, Chiba University School of Medicine, 1-8-1, Inohana, Chiba 260-0856, Japan

**Keywords:** squamous cell carcinoma, actinic keratosis, in situ hybridization, matrix metalloproteinases, extracellular matrix, skin cancer

## Abstract

Matrix metalloproteinase (MMP) plays an important role in extracellular matrix degradation associated with cancer invasion. An expression of MMP-1 (interstitial collagenase), MMP-2 (72-kDa type IV collagenase) and MMP-3 (stromelysin-1) was investigated in squamous cell carcinoma (SCC) and its precancerous condition, actinic keratosis (AK), using in situ hybridization techniques. MMP-1 mRNA was detected in tumour cells and/or in stromal cells in all cases of SCC, four of six AKs adjacent to SCC and four of 16 AKs. MMP-2 and MMP-3 mRNAs were detected in SCC but not in AK. The expression of MMP-3 correlated to that of MMP-1 (*P* = 0.03) localized at the tumour mass and stroma of the invasive area, while MMP-2 mRNA was detected widely throughout the stroma independent of MMP-1 expression. Our results indicated that the expression of MMP-1, -2 and -3 showed different localization patterns, suggesting a unique role of each MMP in tumour progression. Moreover, MMP-1 expression could be an early event in the development of SCC, and AK demonstrating MMP-1 mRNA, might be in a more advanced dysplastic state, progressing to SCC. © 1999 Cancer Research Campaign


*British Journal of Cancer* (1999) **80**(7), 1087–1091
© 1999 Cancer Research Campaign
Article no. bjoc.1998.0468

### Expression of matrix metalloproteinase-1, -2 and -3 in squamous cell carcinoma and actinic keratosis

**R Tsukifuji[1], K Tagawa[2], A Hatamochi[2] and H Shinkai[2]**

[1]Division of Dermatology, National Chiba Hospital, 4-1-2, Tsubakimori Chiba 260-0042, Japan; [2]Department of Dermatology, Chiba University School of Medicine, 1-8-1, Inohana, Chiba 260-0856, Japan

**Summary** Matrix metalloproteinase (MMP) plays an important role in extracellular matrix degradation associated with cancer invasion. An expression of MMP-1 (interstitial collagenase), MMP-2 (72-kDa type IV collagenase) and MMP-3 (stromelysin-1) was investigated in squamous cell carcinoma (SCC) and its precancerous condition, actinic keratosis (AK), using in situ hybridization techniques. MMP-1 mRNA was detected in tumour cells and/or in stromal cells in all cases of SCC, four of six AKs adjacent to SCC and four of 16 AKs. MMP-2 and MMP-3 mRNAs were detected in SCC but not in AK. The expression of MMP-3 correlated to that of MMP-1 ($P = 0.03$) localized at the tumour mass and stroma of the invasive area, while MMP-2 mRNA was detected widely throughout the stroma independent of MMP-1 expression. Our results indicated that the expression of MMP-1, -2 and -3 showed different localization patterns, suggesting a unique role of each MMP in tumour progression. Moreover, MMP-1 expression could be an early event in the development of SCC, and AK demonstrating MMP-1 mRNA, might be in a more advanced dysplastic state, progressing to SCC.

**Keywords:** squamous cell carcinoma; actinic keratosis; in situ hybridization; matrix metalloproteinases; extracellular matrix; skin cancer

Neoplasms invade and metastasize degrading extracellular matrix (ECM), and therefore a large number of proteases involved in the invasion process have been extensively studied in order to understand tumour behaviour. Matrix metalloproteinases (MMPs) constitute a gene family of highly homologous enzymes capable of degrading ECM components, and play crucial roles in tumour progression (Liotta et al, 1991). MMP-1 (interstitial collagenase) degrades types I, II and III collagens, and MMP-2 (72-kDa type IV collagenase) degrades gelatin, types IV, V, VII and X collagens. MMP-3 (stromelysin-1) with a broad substrate specificity, degrades proteoglycans, laminin, fibronectin, gelatin and types III, IV, V and IX collagens (Mauviel et al, 1993). In skin cancer, the expression of MMP-1 and MMP-2 mRNAs has been revealed in basal cell carcinoma (BCC) and squamous cell carcinoma (SCC) (Gray et al, 1992; Pyke et al, 1992). Recent studies have demonstrated that MMP-3 mRNA is expressed in BCC (Majmudar et al, 1994; Tsukifuji et al, 1997).

Although previous studies have suggested that MMP expression is related to tumour invasion (Matrisian et al, 1991; Gray et al, 1992; Pyke et al, 1992; Tsukifuji et al, 1997), the regulatory mechanism for MMPs in tumour tissue has been largely unknown. In tumour progression, the production and activity of MMPs can be changed by cytokines, tissue inhibitors of matrix metalloproteinase (TIMPs) and other proteases derived from tumour cells, fibroblasts and/or inflammatory cells (Liotta et al, 1991; Mauviel et al, 1993). An enormous number of factors should be involved in the extensive ECM degradation of malignant tumour tissue. It is therefore conceivable that the study of carcinoma at early stage or in situ is valuable for understanding the essential roles of MMPs in the multiple steps of tumour progression.

*Received 28 May 1998*
*Revised 19 November 1998*
*Accepted 24 November 1998*

*Correspondence to:* R Tsukifuji

In the present study, we investigated the expression of MMP-1, -2 and -3 in skin cancer, focusing on the early events occurring in invasive tumours. In situ hybridization can identify the cells responsible for the protein and detect the mRNA before its local accumulation. SCC and actinic keratosis (AK), a precancerous condition of SCC, were examined in this study, and the roles of MMPs during tumour progression were discussed.

### MATERIALS AND METHODS

### Tissue samples

Tissue specimens of skin tumours (15 SCCs, six AKs associated with SCC and 16 AKs) were obtained by surgical resection at Chiba University Hospital. The specimens were fixed in 10% buffered formalin immediately after the operation and embedded in paraffin. Four-micrometer tissue sections were mounted on aminoacyl silane-coated glass slides, dried overnight at 42°C and immediately used for in situ hybridization and immunohistochemical examination. The sections stained with haematoxylin and eosin were examined independently by two or more experienced pathologists and dermatologists for histological diagnosis. The patients with SCC were staged according to UICC TNM classification (Sobin et al, 1997).

### RNA probe preparation

RNA probe preparation for MMP-1 and MMP-3 has been described previously (Tsukifuji et al, 1997). A template fragment for MMP-2 was obtained from human fibroblast MMP-2 cDNA (Collier et al, 1988), using the restriction enzyme. Each fragment (the 1016-bp MMP-1 fragment extending from nucleotide 273 to 1289, the MMP-2 fragment from 1353 to 1747, and the 573-bp MMP-3 fragment from 828 to 1401) was subcloned into pGEM 7Z vector (Promega, Madison, WI, USA). Labelled probes were

**Table 1**

| Case No. | MMP-1 | | MMP-3 | | MMP-2 | TNM stage |
|---|---|---|---|---|---|---|
| | T | S | T | S | S | |
| 1 | + | + | – | – | ++ | I |
| 2 | + | ++ | – | – | ++ | I |
| 3 | + | ++ | + | + | ++ | I |
| 4 | + | ++ | + | ++ | ++ | I |
| 5 | + | ++ | – | – | ++ | I |
| 6 | ++ | ++ | + | + | + | I |
| 7 | ++ | +++ | – | + | + | I |
| 8 | + | ++ | – | – | +++ | II |
| 9 | + | ++ | + | + | + | II |
| 10 | ++ | ++ | + | + | ++ | II |
| 11 | + | ++ | + | + | ++ | III |
| 12 | + | +++ | + | ++ | ++ | III |
| 13 | +++ | +++ | – | ++ | +++ | III |
| 14 | +++ | +++ | + | +++ | +++ | III |
| 15 | ++ | +++ | – | ++ | +++ | IV |

T: tumour cells; S: stromal cells.

synthesized by in vitro transcription with SP6 or T7 RNA polymerase using digoxigenin-labelled uridine triphosphate as substrate (Digoxigenin RNA labelling mixture, Boehringer Mannheim, Indianapolis, IN, USA).

### In-situ hybridization

Details of the method have been reported previously (Tsukifuji et al, 1997). Deparaffinized sections were treated with proteinase K (4 μg $ml^{-1}$) at 37°C for 20 min, followed by rinsing in phosphate-buttered saline (PBS). Then the specimens were incubated with chondroitin ABC lyase, 0.02 units $ml^{-1}$ (Seikagaku Kogyo, Tokyo, Japan), in buffer solution (0.1 M Tris–HC1 pH 8.0, 0.03 M sodium acetate, 0.05% bovine serum albumin) at 37°C for 30 min. After rinsing in PBS, the slides were refixed in 4% paraformaldehyde for 20 min, and then dipped in 2 mg $ml^{-1}$ glycine for 15 min and 0.2 N hydrochloric acid for 10 min. Immediately after this pretreatment, the sections were prehybridized in 50% formamide and 2 × standard saline citrate at room temperature for 1 h. They were then covered with the antisense or sense RNA probe in hybridization buffer (50% formamide, 10% dextran sulphate, 1 × Denhardt's solution, 200 μg $ml^{-1}$ yeast tRNA, 600 mM sodium chloride, 1 mM EDTA, 0.25% sodium dodecyl sulphate (SDS), 10 mM Tris–HCl pH 7.6) and incubated at 50°C for 18 h in a humidified chamber. The RNA probes of MMP-1, MMP-2 and MMP-3 were used at concentrations of 400, 700 and 700 ng $ml^{-1}$ respectively. After hybridization, the slides were washed under stringent conditions and treated with RNAase to remove the unhybridized probes. The sections were incubated with 1.5% blocking reagent and anti-digoxigenin antibody conjugated with alkaline phosphatase, 1: 500 (Boehringer Mannheim, Indianapolis, IN, USA) for 1 h at room temperature. After rinsing, labelling was visualized by colour products of Fast Red (BioGenex, San Ramon, CA, USA). Counterstaining was done with methyl green.

The evaluation was based on the mean number of labelled cells per high power field (hpf). A minimum of 8 hpfs and up to 15 hpfs were counted using a ×10 eyepiece and a × 40 objective lens and scored as follows; +++ > 20 cells: ++, 2–20 cells per hpf; +, 0.2–2 cells per hpf; –, < 0.2 cells per hpf.

### Statistical methods

The semi-quantitative scores of in situ hybridization were evaluated using Kruskal–Wallis test or Pearson's correlation coefficient, and $P < 0.05$ was considered significant.

### RESULTS

The results of semi-quantitative evaluation of in situ hybridization are summarized in Table 1.

### The expression of MMP-1, -2 and -3 mRNAs in SCC

The expression of MMP-1 mRNA was detected in all SCC samples. Distinct signals for MMP-1 mRNA were observed in tumour cells and spindle-shaped cells of the stroma (Figure 1A, B). The tumour cells expressing MMP-1 mRNA were mainly located at the invasive area of the neoplasm. Fibroblast-like cells with the hybridization signals were observed mostly around the tumour nests. Generally, MMP-1 mRNA appeared to be more abundant in stromal cells than in tumour cells. Superficially invasive tumours in the early stage already demonstrated moderate signals for MMP-1, and those of deeply invasive ones were remarkably pronounced (Table 1).

The expression of MMP-3 mRNA was detected in 11 of the 15 SCCs. Fibroblast-like cells among the tumour nests and a few neoplastic cells were responsible for the hybridization signals for MMP-3 (Figure 1 C, D).

The expression of MMP-2 mRNA was detected to various degrees in fibroblast-like cells of the stroma in all cases, but was not detected in tumour cells (Table 1). The hybridization signals for MMP-2 were generally weak and scattered throughout the stroma (Figure 1E, F).

The expression of each MMP tended to increase according to the tumour progression but the differences were not significant ($P > 0.1$) in the tumour stages (I, II and III). The signals for MMP-3 were localized at limited areas, where abundant MMP-1 mRNA was usually observed (Figure 2A, B). MMP-3-positive cells correlated with MMP-1-positive ones ($P = 0.03$) although the former were not as many as the latter (Table 1). On the other hand, the expression of MMP-2 did not appear to have any relationship with those of MMP-1 ($P = 0.20$) and MMP-3 ($P = 0.69$) (Figure 1 G–I).

Normal skin demonstrated no distinct signals for the MMP mRNAs examined. For a control study, the signals in serial sections treated with sense probes or RNAase were always compared at the same time. Hybridization with the sense probes showed no specific activity (Figure 1B, D, F) and RNAase treatment before hybridization removed positive reactions to antisense probes.

### The expression of MMP-1 mRNA in AK

MMP-1 mRNA was detected in four of the 16 AK specimens. The signals for MMP-1 were found at a minority of basal keratinocytes showing atypical nuclei and disorderly arrangement. Stromal cells in a dense inflammatory infiltrate were also positive for the MMP-1 probe (Figure 2A, B). In another case, MMP-1 mRNA was expressed by the basal and suprabasal keratinocytes in the epidermis proliferating into the dermis (Figure 2C, D). No distinct signals for MMP-2 and MMP-3 mRNAs were detected in these areas. Secondary changes such as erosion or ulceration were not observed near the positive signals. In AKs the signals for MMP-1 were detected in the anaplastic keratinocytes of hypertrophic epidermis with a dense inflammatory infiltrate in the dermis.

*© 1999 Cancer Research Campaign*

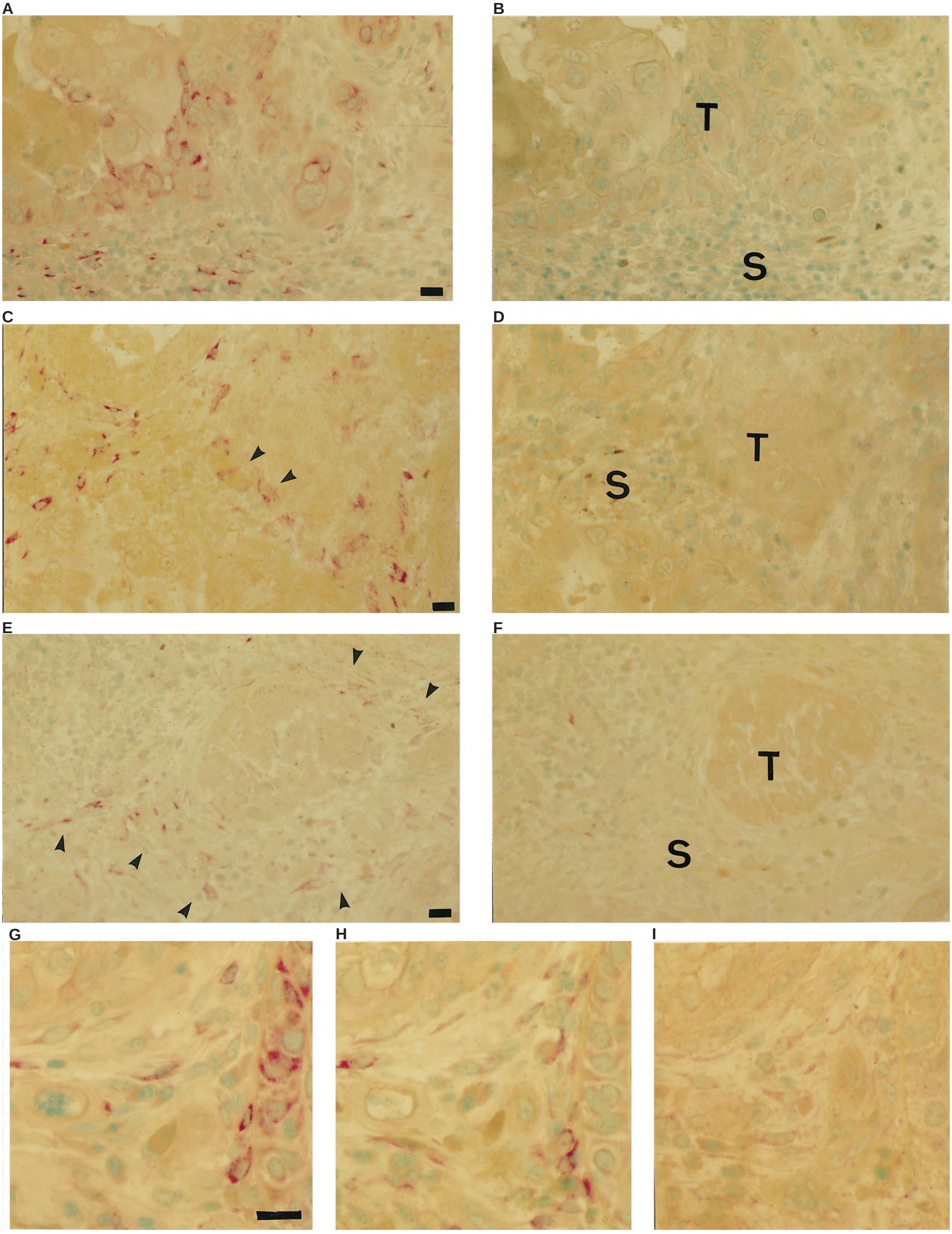

**Figure 1** The expression of matrix metalloproteinases in squamous cell carcinoma by in situ hybridization. The targeted mRNAs are demonstrated by red products. Specific signals for MMP-1 antisense probe are observed in tumour cells and spindle-shaped cells of the stroma (**A**). MMP-1 sense probe shows no specific hybridization signals (**B**). Signals for MMP-3 antisense probe are found in stromal cells and a few tumour cells (arrowheads) (**C**). No signals for MMP-3 sense probe (**D**). The positive cells for MMP-2 antisense probe are scattered throughout the stroma associated with the tumour nests (arrowheads) (**E**) and these cells are negative for MMP-2 sense probe (**F**). T: tumour; S: stroma. In serial sections, abundant signals for MMP-1 (**G**) and moderate for MMP-3 (**H**) are observed while faint signals for MMP-2 are found in the stromal cells (**I**). Scale bars = 20 μm

© 1999 Cancer Research Campaign

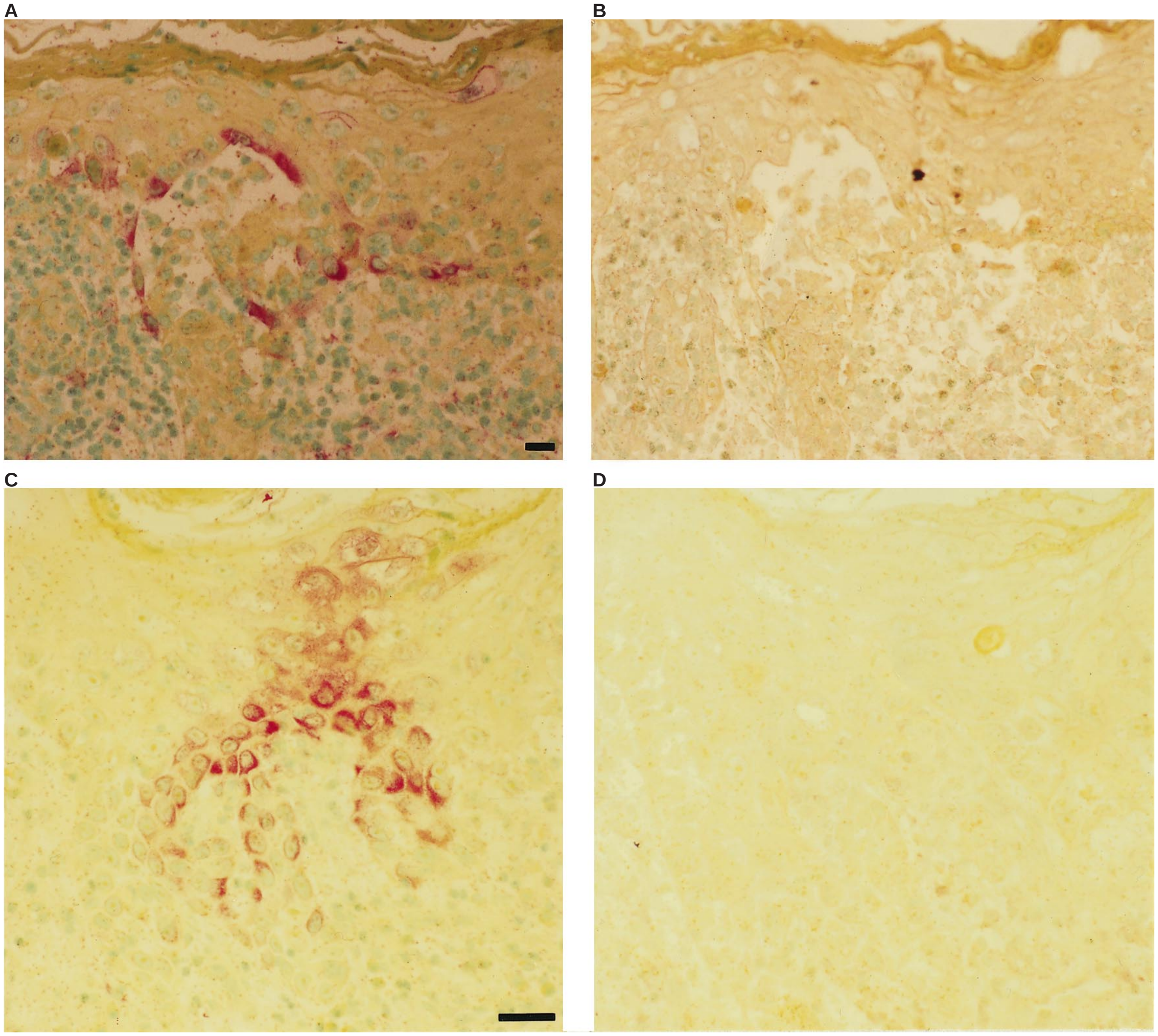

**Figure 2** Hybridization with MMP-1 antisense probe (**A** and **C**) and sense probe (**B** and **D**) in actinic keratosis. MMP-1 mRNA is expressed by the anaplastic cells in the basal layer. Superficial clefts and lacunae are present as a result of secondary acantholysis. The positive signals are also observed in a small number of stromal cells (**A**). No signals for the sense probe (**B**). MMP-1 mRNA is expressed by the keratinocytes of the epidermis extending downwards (**C**). No signals for the sense probe (**D**). Scale bars = 20 μm

The lesions of carcinoma in-situ found adjacent to invasive SCCs were also examined for MMP expression. All such cases examined in the study were found at sun-exposed sites in the elderly and considered to be AK. In four of six AKs accompanied by SCC, expression of MMP-1 mRNA was observed in the keratinocytes and stromal cells. Immunohistochemical staining for MMPs in AK was so weak that the localization of each MMP was not determined.

### DISCUSSION

The most interesting finding in the present study is that MMP-1 mRNA was expressed in AK as well as invasive tumours. In several specimens of AKs and most of AKs associated with SCC, the distinct signals for MMP-1 mRNA were observed in the keratinocytes proliferating downwards. Since there was no erosion or ulceration in the vicinity of MMP-1-positive cells in the AK specimens, MMP-1 mRNA was not induced by acute phase reactions of the wound healing process (Inoue et al, 1995). It has been reported that the activity of MMP-1 is required for keratinocyte migration on a type I collagen matrix (Pilcher et al, 1997). It is possible that atypical keratinocytes expressing MMP-1 in AK could be involved in cell migration associated with the tumour progression.

The expression of MMP-1 mRNA was observed extensively in tumour and stromal cells of SCC, consistent with the previous report (Gray et al, 1992). In basal cell carcinoma (BCC), which is rarely metastatic, MMP-1 mRNA was expressed in stromal cells and detected in relatively large tumours of nodulo-ulcerative type, but not in small tumours of superficial type (Tsukifuji et al, 1997). On the contrary, MMP-1 was produced by tumour and stromal cells from the early stage of SCC and carcinoma in situ. These different patterns of MMP-1 expression might explain the differences in invasive and metastatic behaviour between SCC and BCC.

*© 1999 Cancer Research Campaign*

Although it was suggested that MMP-3 could be significantly involved in the progression of SCC (Matrisian et al, 1991), the expression of MMP-3 mRNA was difficult to detect in SCC by in situ hybridization (Muller et al, 1993). Our study distinctly indicated that MMP-3 mRNA was expressed in SCC specimens which revealed MMP-1 mRNA at high levels. Its localization pattern suggests that its expression may be linked, to some degree, with that of MMP-1. Since MMP-3 mRNA was not detected in AK, MMP-3 seemed to be induced subsequently to MMP-1 during tumour invasion. MMP-1 and MMP-3 promoters contain a 12-O-tetradecanoyl-phorbol-13 acetate (TPA)-responsive element that binds the transcription factor AP-1 (Angel et al, 1987), and the two enzymes may be regulated by similar activating mechanisms.

The expression of MMP-2 mRNA did not correlate well with the SCC stages. MMP-2 mRNA was expressed throughout the stroma around the tumour mass, and this did not suggest a close interaction between tumour and stromal cells. However, our immunohistochemical examination demonstrated that MMP-2 protein appeared to be concentrated at the interface between the tumour mass and stroma (data not shown). As MMP-2 is supposed to be activated at cell surface (Sato et al, 1994), the localization of MMP-2 protein is an interesting issue in the regulation of MMPs.

As for the degradation of type IV collagen, we also searched MMP-9 gene expression, but it was undetectable in tumour cells or stromal cells. In SCC, MMP-9 mRNA seems to be expressed at low levels in small populations of tumour cells (Juarez et al, 1993), and possibly stromal cells (Ståhle-Bäckdahl et al, 1993). High levels of MMP-9 gene expression have been reported in ovarian tumours (Naylor et al, 1994), brain tumours (Nakagawa et al, 1994) and pancreatic tumours, while MMP-1 and MMP-3 were not detected in pancreatic tumours (Gress et al, 1995). In breast carcinoma tissue, MMP-1 was especially abundant in scirrhous carcinoma, containing great amounts of interstitial collagen, compared to papillotubular and solid-tubular carcinoma by immunohistochemical examination (Iwata et al, 1996). These reports suggest that the diversity of MMP gene expression in neoplasms may be related to the components of tumour-associated stroma as well as the origin and invasive behaviour of tumour cells.

Since MMP-3 can activate MMP-1 (Suzuki et al, 1990), its production would enhance tumour invasion into the connective tissue containing abundant interstitial collagen. It was recently reported that skin tumours arising in mice deficient of c-*fos*, a member of the AP-1 family, failed to undergo malignant conversion, and neither MMP-1 nor MMP-3 was induced (Saez et al, 1995). It is interesting and worthy of further investigation to try to determine how malignant tumour transformation is related to ECM-degrading capacity, including the expression of MMP-1 and MMP-3 in skin tumours.

Taken together, our study indicated that MMP-1, -2 and -3 showed different localization patterns in SCC. Abundant MMP-1 mRNA was detected in SCC, and moreover, it was expressed frequently in AK associated with SCC and sometimes in AK. Its expression in AK might be one of the markers for more advanced dysplastic states, progressing to SCC.

### ACKNOWLEDGEMENTS

The authors thank Dr Hidetoshi Ino for his useful advice and Ms Ayako Oikawa for her technical assistance. This work was supported by a Grant-in Aid for Scientific Research (08670947) from the Ministry of Education, Science and Culture of Japan.

### REFERENCES

Angel P, Imagawa M, Chiu R, Stein B, Imbra RJ, Rahmsdorf HJ, Jonat C, Herrlich P and Karin M (1987) Phorbol ester-inducible genes contain a common cis element recognized by a TPA modulated trans-acting factor. *Cell* **9**: 729–739

Collier IE, Wilhelm SM, Eisen AZ, Marmer BL, Grant GA, Seltzer JL, Kronberger A, HC, Bauer EA and Goldberg GI (1988) H-ras oncogene-transformed human bronchial epithelial cells (TBE 1) secrete a single metalloprotease capable of degrading basement membrane collagen. *J Biol Chem* **263**: 6579–6587

Gray ST, Wilkins RJ and Yun K (1992) Interstitial collagenase gene expression in oral squamous cell carcinoma. *Am J Pathol* **141**: 301–306

Gress TM, Müller-Pillasch F, Lerch MM, Friess H, Büchler M and Adler G (1995) Expression and in situ localization of genes coding for extracellular matrix proteins and extracellular matrix degrading proteases in pancreatic cancer. *Int J Cancer* **62**: 407–413

Inoue M, Kratz G, Haegerstrand A and Ståhle-Bäckdahl M (1995) Collagenase expression is rapidly induced in wound-edge keratinocytes after acute injury in human skin, persists during healing, and stops at re-epithelialization. *J Invest Dermatol* **104**: 479–483

Iwata H, Kobayashi S, Iwase H, Masaoka A, Fujimoto N and Okada Y (1996) Production of matrix metalloproteinases and tissue inhibitors of metalloproteinases in human breast carcinomas. *Jpn J Cancer Res* **87**: 602–611

Juarez J, Clayman G, Nakajima M, Tanabe KK, Saya H, Nicolson GL and Boyd D (1993) Role and regulation of expression of 92-kDa type-IV collagenase (MMP-9) in 2 invasive squamous-cell carcinoma cell lines of the oral cavity. *Int J Cancer* **55**: 10–18

Liotta LA, Steeg PS and Stetler-Stevenson WG (1991) Cancer metastasis and angiogenesis: an imbalance of positive and negative regulation. *Cell* **64**: 327–336

Majmudar G, Nelson BR, Jensen TC and Johnson TM (1994) Increased expression of matrix metalloproteinase-3 (stromelysin-1) in cultured fibroblasts and basal cell carcinomas of nevoid basal cell carcinoma syndrome. *Mol Carcinog* **11**: 29–33

Matrisian LM, McDonnell S, Miller DB, Navre M, Seftor EA and Hendrix MJC (1991) The role of the matrix metalloproteinase stromelysin in the progression of squamous cell carcinomas. *Am J Med Sci* **302**: 157–162

Mauviel A (1993) Cytokine regulation of metalloproteinase gene expression. *J Cell Biochem* **53**: 288–295

Muller D, Wolf C, Abecassis J, Millon R, Engelmann A, Bronner G, Rouyer N, Rio M-C, Eber M, Methlin G, Chambon P and Basset P (1993) Increased stromelysin 3 gene expression is associated with increased local invasiveness in head and neck squamous cell carcinomas. *Cancer Res* **53**: 165–169

Naylor MS, Stamp GW, Davies BD and Balkwill FR (1994) Expression and activity of MMPs and their regulators in ovarian cancer. *Int J Cancer* **58**: 50–56

Nakagawa T, Kubota T, Kabuto M, Sato K, Kawano H, Hayakawa T and Okada Y (1994) Production of matrix metalloproteinases and tissue inhibitor of metalloproteinase-1 by human brain tumors. *J Neurosurg* **81**: 69–77

Pilcher BK, Dumin JA, Sudbeck BD, Krane SM, Welgus HG and Parks WC (1997) The activity of collagenase-1 is required for keratinocyte migration on a type I collagen matrix. *J Cell Biol* **137**: 1455–1457

Pyke C, Ralfkiær E, Huhtala P, Hurskainen T, Dano K and Tryggvason K (1992) Localization of messenger RNA for *M*r 72 000 and 92 000 type IV collagenases in human skin cancers by in situ hybridization. *Cancer Res* **52**: 1336–1341

Saez E, Rutberg SE, Mueller E, Oppenheim H, Smoluk J, Yuspa SH and Spiegelman BM (1995) c-*fos* is required for malignant progression of skin tumors. *Cell* **82**: 721–732

Sato H, Takino T, Okada Y, Cao J, Shinagawa A, Yamamoto E and Seiki M (1994) A matrix metalloproteinase expressed on the surface of invasive tumor cells. *Nature* **370**: 61–65

Sobin LH and Wittekind Ch (1997) UICC TNM *Classification of Malignant Tumours*, 5th edn, Sobin LH, Wittekind Ch (eds), pp. 115–117. Wiley-Liss Inc: New York

Ståhle-Bäckdahl M and Parks WC (1993) 92-kd gelatinase is actively expressed by eosinophils and stored by neutrophils in squamous cell carcinoma. *Am J Pathol* **142**: 995–1000

Suzuki K, Enghild JJ, Morodomi T, Salvesen G and Nagase H (1990) Mechanisms of activation of tissue procollagenase by matrix metalloproteinase 3 (stromelysin). *Biochemistry* **29**: 10261–10270

Tsukifuji R, Sakai T, Hatamochi A and Shinkai H (1997) Gene expression of matrix metalloproteinase 1 (interstitial collagenase) and -3 (stromelysin-1) in basal cell carcinoma by in situ hybridization using chondroitin ABC lyase. *Histochem J* **29**: 1–7

© 1999 Cancer Research Campaign 
